# Optimization of donor structure enhances the generation of cloned goats with high expression of human butyrylcholinesterase by CRISPR/Cas9

**DOI:** 10.3389/fbioe.2025.1633553

**Published:** 2025-11-10

**Authors:** Yunpeng Wu, Jianhua Zheng, Jingqing Chen, Tianqi Sun, Tianqi Luan, Yan Li, Yunzhi Fa, Yefeng Qiu, Rui Zhang

**Affiliations:** 1 Academy of Military Medical Sciences, Beijing, China; 2 Laboratory of Advanced Biotechnology, Beijing, China; 3 College of Life Science and Technology, Mudanjiang Normal University, Mudanjiang, China

**Keywords:** CRISPR/Cas9, donor structure, HDR, butyrylcholinesterase, goat

## Abstract

**Introduction:**

Human butyrylcholinesterase (hBChE) is a promising bioscavenger against organophosphorus (OP) nerve agents and pesticides. However, low homology-directed repair (HDR) efficiency in CRISPR/Cas9-mediated genome editing limits precise transgene integration in large animals.

**Methods:**

To improve HDR, we optimized donor structure for targeted integration of hBChE into the goat *FGF5* locus using CRISPR/Cas9. Correctly edited goat fibroblast clones were identified by PCR and sequencing. A homozygous clone with reverse-oriented integration was used as a donor for somatic cell nuclear transfer (SCNT), followed by embryo transfer. Offspring were analyzed for genomic integration and transgene expression.

**Results:**

Reverse-oriented donors significantly enhanced HDR efficiency compared to forward designs, with validation at the pig *RAG1* locus. Edited cells stably expressed recombinant hBChE (rhBChE) and showed increased resistance to OP pesticides. SCNT produced a cloned goat expressing high rhBChE levels in the skin.

**Discussion:**

Optimizing donor structure improves precise genome editing efficiency and enables robust generation of transgenic goats. This strategy advances CRISPR/Cas9-based bioreactor development for scalable production of therapeutic proteins.

## 1 Introduction

Human butyrylcholinesterase (hBChE) is a non-specific cholinesterase capable of hydrolyzing a variety of choline esters and is widely distributed in tissues such as the liver, brain, heart, and plasma (Johnson and Moore, 2012). Broomfield et al. (1991) found that the injection of BChE purified from horse serum into monkeys could effectively counteract the toxic effects of organophosphorus nerve agents (OPNAs), highlighting the potential of BChE in chemical defense. Subsequent studies have further confirmed, through *in vitro* and *in vivo* experiments in rodents, miniature pigs, and non-human primates, that hBChE can effectively prevent the OPNAs poisoning (Lenz et al., 2005; Saxena et al., 2011a; Saxena et al., 2011b; Rice et al., 2016; Reed et al., 2017; Rosenberg and Saxena, 2020). Notably, hBChE administration significantly improved survival rates following VX poisoning (Rice et al., 2016; Mann et al., 2018). At present, hBChE is regarded as the most promising biological scavenger due to its long biological half-life, safety, high reactivity and universality (Johnson and Moore, 2012). However, as a stoichiometric biological scavenger, hBChE requires a high dose for therapeutic efficacy, and serum-derived purification cannot meet the demands of large-scale production. Recombinant human butyrylcholinesterase (rhBChE) has been successfully expressed in yeast, CHO cells, tobacco and rice, but the recombinant enzymes often lack therapeutic efficacy (Xing et al., 2021).

Transgenic animal mammary gland bioreactors have emerged as efficient platforms for producing medicinal proteins (Schmidt, 2006; Amiri Yekta et al., 2013; Cui et al., 2015), and biomaterials (Xu et al., 2007). Compared with traditional methods, mammary gland bioreactors offer high productivity, low cost, humanized glycosylation profiles, low energy consumption, environmental sustainability, and minimal disruption to animal physiology (Hennighausen, 1990; Houdebine, 2009). Several functional proteins have been produced using this technology, including recombinant human coagulation factors (Amiri Yekta et al., 2013), recombinant human antithrombin (ATRyn) (Schmidt, 2006), recombinant human lactoferrin (Goldman et al., 2012), monoclonal antibodies (Zhang et al., 2009; Zhang et al., 2012), recombinant human α-lactalbumin (Wang et al., 2008), recombinant human lysozyme (Maga et al., 2006), etc. Notably, ATRyn, expressed in milk of transgenic goats, was approved by the US FDA in 2009 (Adiguzel et al., 2009), and C1 esterase inhibitor produced in transgenic rabbit milk was approved by the US FDA in 2014 (Varga and Farkas, 2011). Huang et al. (2007) demonstrated the feasibility of mass-producing functional rhBChE in the milk of transgenic mice and goats. However, random integration of exogenous genes into the genome may disrupt target gene expression (Aoyama et al., 2005; Gui et al., 2012).

The development of CRISPR/Cas9 genome editing technology has significantly improved in the efficiency of targeted exogenous genes integration and the generation of transgenic animals. The CRISPR/Cas9 system induces targeted DNA double-strand breaks (DSBs), triggering endogenous repair mechanisms that enable precise insertion, deletion, or replacement of DNA sequences (Li et al., 2019). Nevertheless, DSBs repair pathways mainly include classical nonhomologous end joining (cNHEJ) or homology-directed repair (HDR) (Scully et al., 2019). Since HDR is mainly active during the S and G2 phases and requires a homologous repair template, its frequency is substantially lower than that of cNHEJ (Scully et al., 2019), limiting the application of precise editing for transgenic animal producing. Therefore, the low efficiency of HDR remains a major bottleneck for CRISPR/Cas9-mediated precise genome editing.

Various approaches have been employed to overcome the low efficiency of HDR in genome editing. At present, the main strategies to improve HDR efficiency include: screening for highly efficient sgRNA targets, optimizing donor templates, and regulating DSBs repair pathways. Since HDR occurrence depends on the formation of DSBs, enhancing the efficiency of the CRISPR/Cas9 system in generating DSBs at target sites in crucial for improving HDR outcomes. However, the ability of Cas9 to induce DSBs varies greatly at different genomic loci, potentially due to the Cas9-sgRNA complex’s sequence preferences (Allen et al., 2018; Chakrabarti et al., 2019). Studies have shown that a high GC content in the sgRNA sequence can reduce its activity (Wang et al., 2014). In addition, variations in the protospacer adjacent motif (PAM) sequence, particularly the third base, influence Cas9 efficiency. For instance, the presence of cytosine (CGG) at the variable position of the PAM sequence enhances Cas9 activity, whereas thymine (TGG) negatively impacts it (Safari et al., 2017). Moreover, the physical form of the donor template (linear vs. circular) and the length of the homology arms are key factors affecting the efficiency of HDR. Auer et al. (2014) reported that the linearized donor templates achieve significantly higher HDR rates than circular plasmids. Wang et al. (2023) showed that homology arms (HAs) of 800–1,000 bp result in optimal HDR efficiency in goat fetal fibroblasts. It has also been reported that single-stranded DNA (ssDNA) donors or single-stranded oligonucleotide (ssODN) donor exhibit higher HDR efficiencies and lower cytotoxicity compared to double-stranded DNA (dsDNA) templates (Richardson et al., 2016; Ma et al., 2017). Furthermore, small molecules can enhance HDR by either inhibiting the cNHEJ pathway, promoting the HDR pathway, or arresting the cell cycle in the G2/M phase. Chu et al. (2015) reported that inhibiting key cNHEJ factors such as KU70 or DNA ligase IV could increase HDR efficiency by 4- to 5-fold. When E1B55K and E4orf6 were co-expressed with Cas9, HDR efficiency increased by up to 8-fold while nearly eliminating cNHEJ activity in human and mouse cell lines. Similarly, Li et al. (2023) demonstrated that small molecules disrupting the p53-MDM2 interaction, such as RITA, Nutlin3, and CTX1, induced G2/M arrest and significantly improved CRISPR-Cas9-mediated HDR efficiency in sheep fetal fibroblasts by 1.43- to 4.28-fold.

As a negative regulator of hair (wool) growth, fibroblast growth factor (*FGF5*) has been shown to influence hair length in various species, including sheep (Li et al., 2017; Zhang et al., 2020), goats (Wang et al., 2015; Wang et al., 2016), donkeys (Legrand et al., 2014), cats (Drögemüller et al., 2007; Kehler et al., 2007), dogs (Housley and Venta, 2006; Dierks et al., 2013), and even humans (Higgins et al., 2014). Multiple studies have demonstrated the feasibility and reproducibility of precise *FGF5* editing in goats and sheep (Wang et al., 2015; Wang et al., 2016; Li et al., 2017; Zhang et al., 2020), and successful expression of target gene has been achieved by inserting different exogenous genes into the *FGF5* locus (Hu et al., 2021; Wang et al., 2023). Thus, the *FGF5* gene serves as a feasible and reliable target site for precise knock-in of an rhBChE expression cassette, enabling the generation of goats overexpressing rhBChE using CRISPR/Cas9-mediate genome editing.

In summary, this study aimed to achieve rhBChE overexpression in goats using CRISPR/Cas9 technology. By optimizing donor structure, we improved HDR efficiency and enhanced positive clone selection providing a practical approach for the efficient production of cloned goats overexpressing rhBChE, which can serve as organophosphate antidote bioreactors.

## 2 Materials and methods

### 2.1 Ethics statement

All animal experimental protocols in this study were approved and carried out in strict accordance with guidelines for the Animal Care and Use Committee at Academy of Military Medical Sciences (approval number IACUC-DWZX-2023–301). The study strictly adhered to the Guidelines for the Care and Use of Laboratory Animals issued by the National Institutes of Health.

### 2.2 Plasmids and donor templates construction

Three sgRNAs targeting the goat *FGF5* locus were selected in this study. PX458 plasmids, containing Cas9 and U6-sgRNA co-expression backbones, were obtained from Addgene and linearized using BbsI-HF (NEB, United States). Complementary sgRNA oligos were synthesized, annealed, and individually ligated into the linearized PX458 backbone vector to construct PX458-sgRNA plasmids. Plasmids were purified using the Endofree Plasmid Maxi Kit (Qiagen, Germany).

A PX458-sgRNA plasmid targeting the pig *RAG1* gene was constructed using the method described above. Subsequently, the EGFP reporter gene in the PX458 backbone was replaced with EBFP to generate the PX458-EBFP-sgRNA plasmid.

Forward and reverse donor templates for HAs-CMV-mCherry-polyA, HAs-EF1α-EGFP-polyA, and HAs-CMV-FLAG-hBChE-polyA were constructed using the In-Fusion Snap Assembly Master Mix (Takara, Japan). For forward templates, left and right homology arms (approximately 1 kb each) were combined within the 5′and 3′ends of CMV-mCherry-polyA, EF1α-EGFP-polyA, or CMV-FLAG-hBChE-polyA, respectively. Conversely, reverse templates were assembled by combining these homology arms with the 3′and 5′ends of the respective constructs. The hBChE gene was obtained by extracting RNA from 293T cells using the MiniBEST Universal RNA Extraction Kit (Takara, Japan), followed by cDNA synthesis with the PrimeScript RT Reagent Kit with gDNA Eraser (Takara, Japan). The complete open reading frame (ORF) regions of hBChE cDNA was amplified using PrimeSTAR GXL Premix Fast, Dye plus (Takara, Japan). The HAs-P2A-mCherry-TFB donor templates were also constructed using the In-Fusion Snap Assembly Master Mix (Takara, Japan). All donor templates were ligated into the linearized vectors using the In-Fusion Snap Assembly Master Mix (Takara, Japan). Plasmids were purified with the Endofree Plasmid Maxi Kit (Qiagen, Germany), and donor DNA fragments were amplified using PrimeSTAR GXL Premix Fast, Dye plus (Takara, Japan).

### 2.3 Cell lines culture and transfection

Goat fibroblast cells (gFCs) were cultured in DMEM/F12 (Gibco, United States) supplemented with 10% fetal bovine serum (FBS, Gibco, United States) and 1% penicillin/streptomycin (Gibco, United States) at 37 °C in a 5% CO_2_ atmosphere. Pig fetal fibroblast cells (PFFs) were cultured in MSC medium (ScienCell, United States) supplemented with 10% FBS and 1% penicillin/streptomycin (Gibco, United States) at 38.5 °C in a 5% CO_2_ atmosphere. Upon reaching approximately 80%–90% confluency, the gFCs and PFFs were trypsinized for subsequent passage or cryopreservation.

For transfection, 2.5 × 10^6^ cells were mixed with 10 μg PX458-sgRNA plasmid and 5 μg of donor template in 100 μL of nucleofector solution. Transfection was performed using the Amaxa Basic Nucleofector Kit for Primary Fibroblasts and the Nucleofector 2b Device (Lonza, Switzerland) with program A-033. Post-transfection, cells were transferred to 6-well plates for further culture.

### 2.4 HDR efficiency assessment

To evaluate HDR efficiency, PX458-sgRNA plasmids and HAs-P2A-mCherry-TFB donor DNA fragment were co-transfected into gFCs. After 48 h of cultivation, cells were stained with the Zombie NIR Fixable Viability Kit (BioLegend, United States) and resuspended in PBS containing 3% FBS. mCherry-positive (mCherry^+^) viable cells were quantified using a BD FACSAria II flow cytometer.

For evaluating both forward and reverse knock-in efficiencies, PX458-sgRNA plasmids were co-transfected into gFCs with either forward or reverse HAs-CMV-mCherry-polyA donor templates. After 48 h, cells were stained and resuspended in complete DMEM/F12 medium with 20% FBS and 1% penicillin/streptomycin. 2000 viable cells expressing both double EGFP and mCherry were then sorted into each well of a 6-well plate using a BD FACSAria II flow cytometer and cultured for 12 days. mCherry^+^ viable cells were quantified using a BD FACSAria II flow cytometer.

Similarly, PX458-EBFP-sgRNA plasmids were co-transfected into PFFs with either forward or reverse HAs-EF1α-EGFP-polyA donor templates. After 48 h, cells were stained with the 7-AAD and resuspended in complete MSC medium with 20% FBS and 1% penicillin/streptomycin. 5,000 viable cells expressing both double EBFP and EGFP were then sorted into each well of a 6-well plate using a BD FACSAria II flow cytometer and cultured for 14 days. EGFP-positive (EGFP ^+^) viable cells were quantified using a BD FACSAria II flow cytometer.

### 2.5 Identification of mutation types in the pig *RAG1* gene

PX458-EBFP-sgRNA plasmids were transfected into PFFs. Single EBFP-positive (EBFP^+^) cells were sorted into 96-well plates using a BD FACSAria II flow cytometer and cultured for 12 days to allow colony formation. Individual cell colonies were lysed in a buffer containing 4% Tris-HCl (1 M, pH = 8.0), 0.9% TritonX-100, 0.9% NP-40, and 0.4 mg/mL proteinase K (all from Solarbio, China). Lysis was performed at 65 °C for 30 min, followed by heating at 95 °C for 15 min, and cooling to 4 °C. Lysates were directly used for PCR identification with PrimeSTAR GXL Premix Fast, Dye plus (Takara, Japan). Primers are listed in Supplementary Table S1. PCR products were subsequently cloned into the pEASY-Blunt Zero Cloning Vector (TransGen, China) and verified by Sanger sequencing.

### 2.6 Generation of cell clones with genomic integration of the hBChE gene

To establish hBChE knock-in cell lines, PX458-sgRNA plasmids were co-transfected into gFCs with either forward or reverse HAs-CMV-FLAG-hBChE-polyA donor templates. Single EGFP^+^ were sorted into 96-well plates using a BD FACSAria II flow cytometer and cultured for 12 days to allow colony formation. Cell clones were lysed using the method described above, and the resulting lysates were used directly as templates for PCR amplification. Primers P1 and P2 detected forward knock-in hBChE clones; N1 and N2 detected reverse knock-in hBChE clones. Primer PN determined zygosity. All primers are listed in Supplementary Table S2. PCR products were analyzed on a 1% agarose gel and visualized using a ChemiDoc imaging system (Bio-Rad, United States). Positive PCR products were subsequently cloned into the pEASY-Blunt zero-cloning vector (TransGen, China) and confirmed by Sanger sequencing.

### 2.7 Measurements of hBChE gene expression by quantitative real-time PCR

Total RNA was extracted using TRIzol reagent (Mei5bio, China) and subsequently reverse-transcribed into cDNA using the PrimeScript RT Kit with gDNA Eraser (Takara, Japan). rhBChE mRNA expression was quantified using primer Q1 and TB Green Premix Ex Taq II (Takara, Japan) on a CFX96 Real-Time System (Bio-Rad, United States), with β-Actin serving as the reference gene. Relative expression levels were analyzed using the 2^-ΔΔCt^ method. Primer sequences in this study are listed in Supplementary Table S3.

### 2.8 Immunofluorescence analysis

hBChE-positive (hBChE+) and wild-type (WT) gFCs were cultured in 24-well plates, fixed with 4% paraformaldehyde tissue fixation solution (Biosharp, BL539A, China) for 15 min, permeabilized with 0.1% Triton X-100 (diluted from 10% sterile stock in PBS; Beyotime, ST797, China) for 10 min, and blocked with 5% bovine serum albumin blocking buffer (Solarbio, SW3015, China) for 60 min. Cells were incubated overnight at 4 °C with Anti-Butyrylcholinesterase antibody (Abcam, ab236577, United Kingdom) diluted 1:1,000 in universal antibody diluent (NCM Biotech, WB500D, China), followed by an incubation with CoraLite488-Conjugated Goat Anti-Rabbit IgG (H + L) (Proteintech, China), diluted at a ratio of 1:1,000 for 60 min in the dark. Nuclei were stained with DAPI solution (Solarbio, C0065, China) for 10 min. Visualization was performed using an Olympus IX71 microscope.

### 2.9 Western blot analysis

Total proteins were extracted from gFCs and goat skin tissue using the Minute Total Protein Extraction Kit for Animal Cultured Cells/Tissues (Inventbiotech, United States). Protein concentrations were then determined with Pierce BCA Protein Assay Kits (Thermo Fisher Scientific, United States). Equal amounts of protein samples were separated on 4%–12% SDS-PAGE gels (Huaxingbio, China) and transferred to PVDF membranes (Thermo Fisher Scientific, United States). Membranes were blocked with 5% Difco skim milk (BD Biosciences, United States) for 60 min at room temperature. For rhBChE detection, membranes were incubated overnight at 4 °C with Recombinant Anti-Butyrylcholinesterase antibody (1:1,000; Abcam, ab151554, United Kingdom) and HRP-conjugated GAPDH antibody (1:10,000; Proteintech, China) as a loading control. After washing, HRP-conjugated Goat Anti-Rabbit IgG (H + L) (1:5,000; Proteintech, China) was applied for 60 min at room temperature to detect rhBChE. For FLAG-tagged protein detection, membranes were incubated overnight at 4 °C with HRP Anti-DDDDK tag antibody (1:1,000; Abcam, ab49763, United Kingdom) and HRP-conjugated Beta Actin antibody (1:5,000; Proteintech, China). Protein bands were detected using an ECL chemiluminescence detection system (Thermo Fisher Scientific, United States) and visualized with a ChemiDoc imaging system (Bio-Rad, United States).

### 2.10 Evaluation of hBChE-Positve cells viability exposed to organophosphorus pesticides

WT gFCs were stained using the Zombie NIR Fixable Viability Kit (BioLegend, United States), and 3,000 viable cells were sorted into each well of a 96-well plate using a BD FACSAria II flow cytometer. These cells were cultured in DMEM/F12 complete medium (Gibco, United States) with varying concentrations of organophosphorus pesticides (OPPs, Aladdin, United States) for 24 h at 37 °C in a 5% CO_2_ incubator. Cell viability was assessed using the CCK-8 Cell Proliferation and Activity Detection Kit (Mei5bio, China), and the median lethal dose (LD_50_) of OPPs was determined.

Subsequently, both WT gFCs and hBChE^+^ gFCs were stained and sorted as above, then cultured in medium containing OPPs at the LD_50_ concentration. After 24 h, cell morphology was observed under an Olympus IX71 microscope, and viability was compared using the CCK-8 assay.

### 2.11 Off-target detection

To evaluate potential off-target effects of the CRISPR-Cas9 system *in vivo*, candidate off-target loci were identified using Cas-OFFinder. Selected loci were amplified by PCR and subcloned into pEASY-Blunt zero-cloning vector (TransGen, China) for validation through Sanger sequencing. Primer sequences are listed in Supplementary Table S4.

### 2.12 Somatic cell nuclear transfer and pregnancy diagnosis

Mature oocytes were collected from healthy female donor laoshan dairy goats and cultured in TCM199 medium (Sigma-Aldrich, United States) supplemented with 2% FBS (Gibco, United States). The oocytes were incubated in TCM199 medium (Sigma-Aldrich, United States) supplemented 5 μg/mL cytochalasin B (CB, Sigma-Aldrich, United States) and 5 μg/mL Hoechst 33,342 (Beyotime, China) for 10 min, followed by enucleation using micromanipulation techniques. Subsequently, hBChE^+^ gFCs were injected into the enucleated oocytes and fused using the ECM2001 Electrocell Manipulator (BTX Inc, United States). The embryos were cultured in TCM199 medium containing 10 μg/mL Cycloheximide (Sigma-Aldrich, United States) and 5 μg/mL CB for an additional 5 h, then transferred to developmental medium for further cultivation and embryo transfer tests. Two months after embryo transfer, pregnancy rates in recipient goats were assessed by ultrasonography.

### 2.13 Statistical analysis

The data were analyzed using Student’s t-test and One-way ANOVA. Results are presented as mean ± standard deviation (SD). Statistical significance was determined as follows: *, *p* < 0.05; **, *p* < 0.01; ***, *p* < 0.001; ****, *p* < 0.0001.

## 3 Results

### 3.1 sgRNA3 of *FGF5* exhibits higher HDR efficiency in goat fibroblast cell line

**FIGURE 1:**
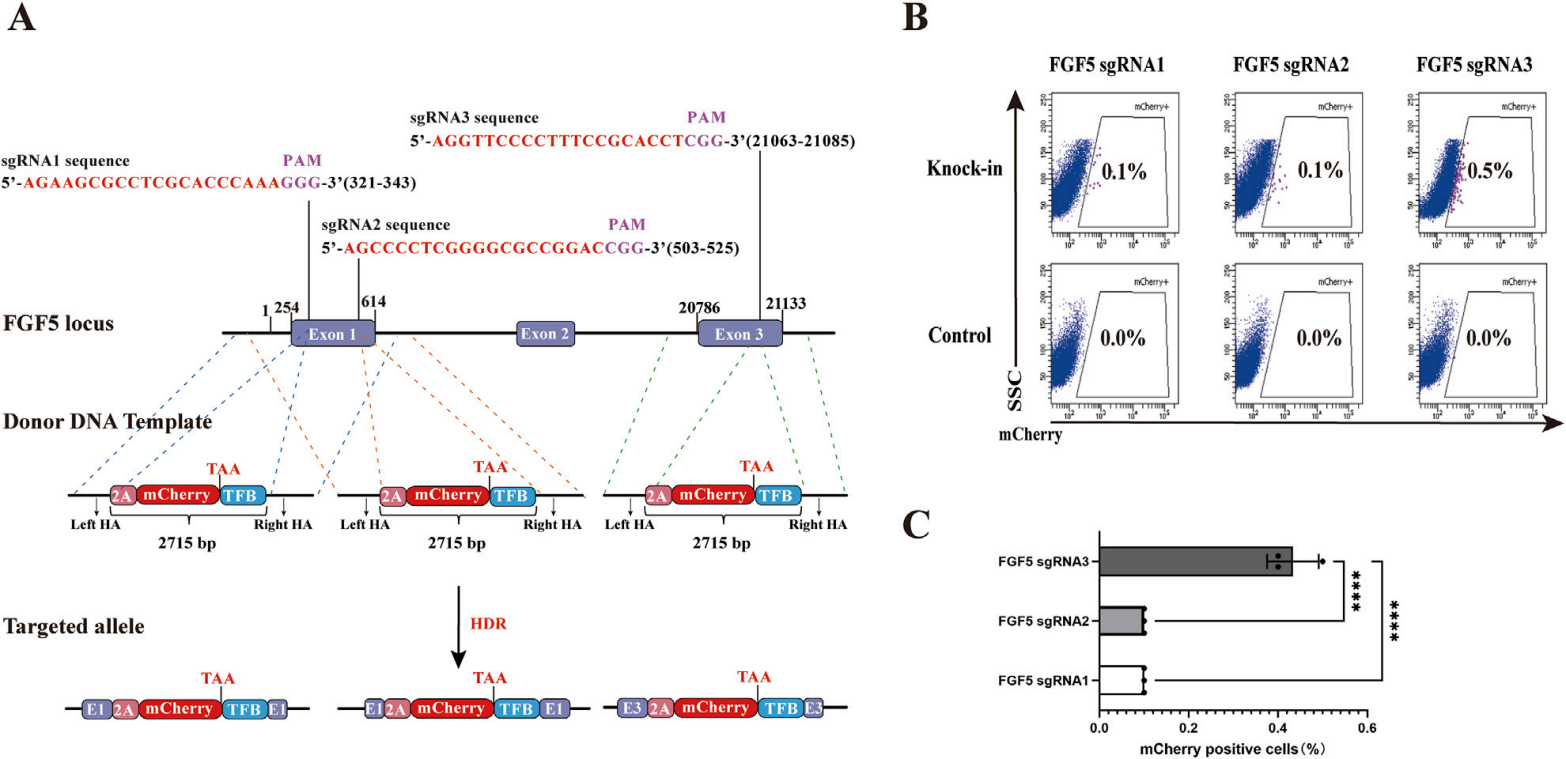
Effects of different sgRNAs targeting the goat *FGF5* locus on HDR efficiency. **(A)** Schematic illustration of donor template design (HAs-P2A-mCherry-TFB) targeting three different sgRNA sites. TFB includes T2A. three FLAG tags, and the hBChE gene. Schematic representation of HAs-P2A-mCherry-TFB donor template structures: pink for P2A, red for mCherry, and blue for TFB. Homology arms are approximately 1 kb in length for all constructs. The schematic is not drawn to scale; variations in visual arm length reflect differences in sgRNA target positions and are for illustrative clarity. **(B)** Flow Cytometry results showing percentages of mCherry^+^ cell after transfection. **(C)** Quantitative comparison of HDR efficiencies among the three sgRNAs. Statistical significance was determined using one-way ANOVA: ****p < 0.0001, n = 3.

The limited HDR efficiency poses a significant challenge for the establishment of genome editing animal models. In this study, we evaluated the HDR efficiency of three sgRNAs targeting the goat *FGF5* locus to identify the one with the highest HDR rate. Three types of homology arms (HAs)-linked P2A-mCherry-TFB fluorescent reporter donor templates were constructed, each targeting one of the three sgRNA sites (Figure 1A). The “TFB” sequence, consisting of T2A, 3X FLAG, and the hBChE gene, was included to extend the total length of the donor template, ensuring comparable sizes between the P2A-mCherry-TFB and CMV-FLAG-hBChE-polyA constructs. The HAs-P2A-mCherry-TFB system enabled the fusion of *FGF5* and mCherry through the P2A translational skipping peptide, allowing the expression of mCherry under the control of the endogenous promoter of *FGF5*. For the knock-in experiments, transfection with PX458-sgRNA plasmids alone served as the negative control. As an experimental control, PX458-sgRNA plasmids were co-transfected with the HAs-P2A-mCherry-TFB donor DNA fragment into gFCs. After 48 h of cultivation, flow cytometry analysis revealed that the proportion of mCherry^+^ viable cells in the sgRNA3 group reached 0.5%, significantly higher than in the other groups (Figures 1B,C). Therefore, sgRNA3 exhibited the highest HDR efficiency among the three candidates.

### 3.2 Reverse knock-in method shows higher HDR efficiency

In CRISPR/Cas9-mediated genome editing, targeted knock-in of an expression vector at a specific genomic locus can be performed either in the forward orientation, aligning with the transcriptional direction of the target gene (from 5′to 3′), or alternatively in the reverse orientation, inserting the expression vector from the 3′to 5′direction relative to the target gene’s orientation. However, few studies have explored the reverse knock-in method.

**FIGURE 2:**
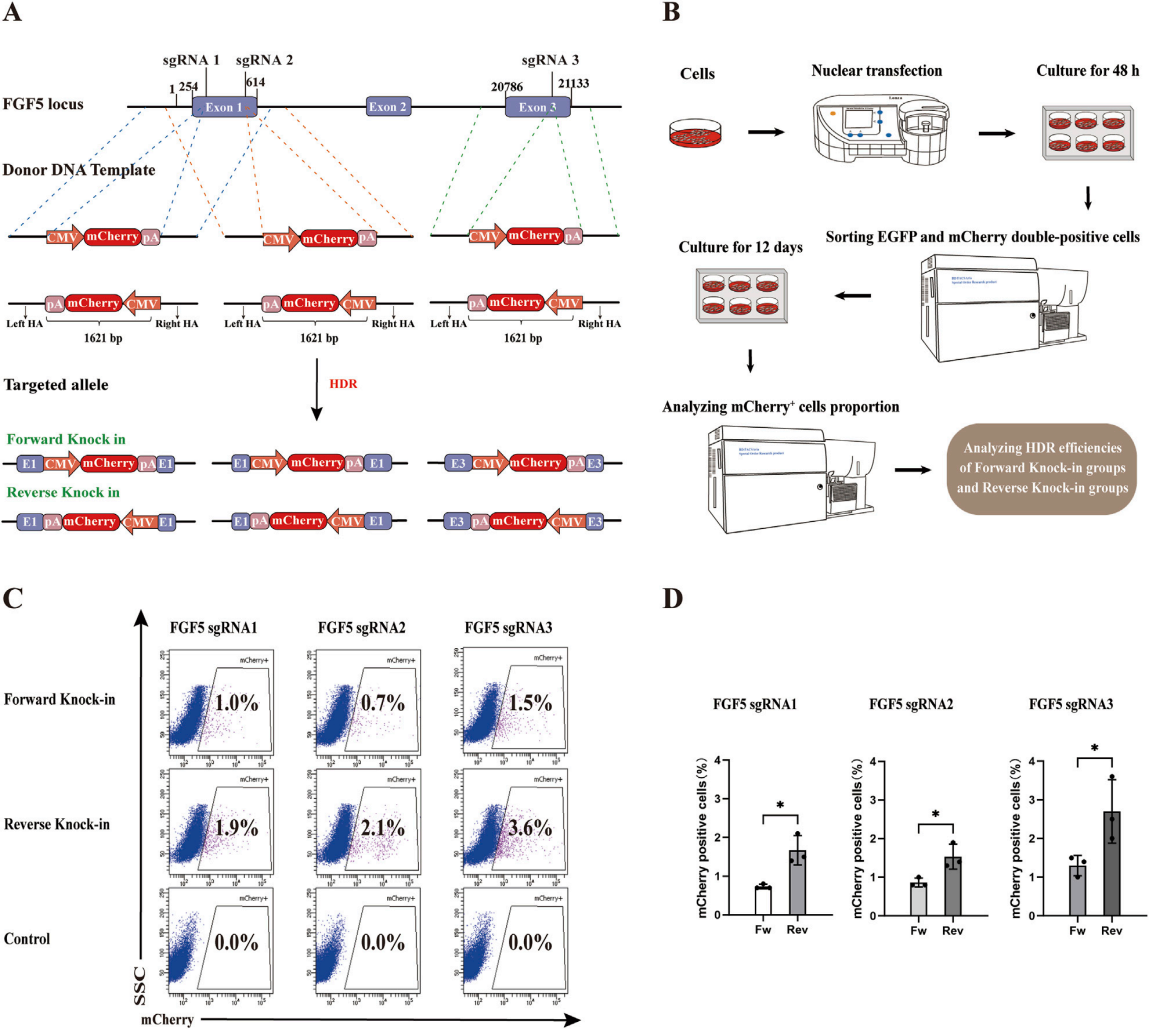
Comparison of HDR efficiency between forward and reverse knock-in methods in the goat genome. **(A)** Schematic diagrams of donor template design (HAs-CMV-mCherry-polyA) for both forward and reverse knock-in at three sgRNA sites in the goat *FGF5* locus. CMV: cytomegalovirus promoter; pA: bovine growth hormone polyadenylation signal (bGH polyA signal). **(B)** Workflow for assessing HDR efficiency following co-transfection and selection. **(C)** Flow Cytometry results showing mCherry^+^ cell percentages in forward and reverse knock-in groups. **(D)** Quantitative comparison of HDR efficiencies between forward and reverse knock-in groups. Statistical significance was determined using Student’s t-test: *p < 0.05, n = 3.

To evaluate the efficiency of forward versus reverse knock-in methods, we selected three sgRNAs targeting the *FGF5* locus and constructed corresponding forward and reverse donor templates (HAs-CMV-mCherry-polyA) (Figure 2A). In the forward knock-in groups, PX458-sgRNA plasmids were co-transfected with forward donor templates into gFCs, while in the reverse knock-in groups, PX458-sgRNA plasmids were co-transfected with reverse donor templates. PX458-sgRNA plasmids co-transfected with CMV-mCherry-polyA fragments lacing homology arms served as negative controls. 48 h after transfection, 2000 double-positive (EGFP and mCherry^+^) cells were sorted from each group and cultured for 12 days to eliminate transient expression and allow degradation of non-integrated donor templates (Figure 2B). Flow cytometry analysis revealed that, for all three sgRNAs, the proportion of mCherry^+^ cells was significantly higher in the reverse knock-in groups compared to the forward knock-in groups (Figures 2C,D). Among them, sgRNA3 exhibited the highest HDR efficiency in both orientations. These results demonstrate that the reverse knock-in method achieves higher HDR efficiency than the forward knock-in method across all tested target sites within the *FGF5* locus.

**FIGURE 3:**
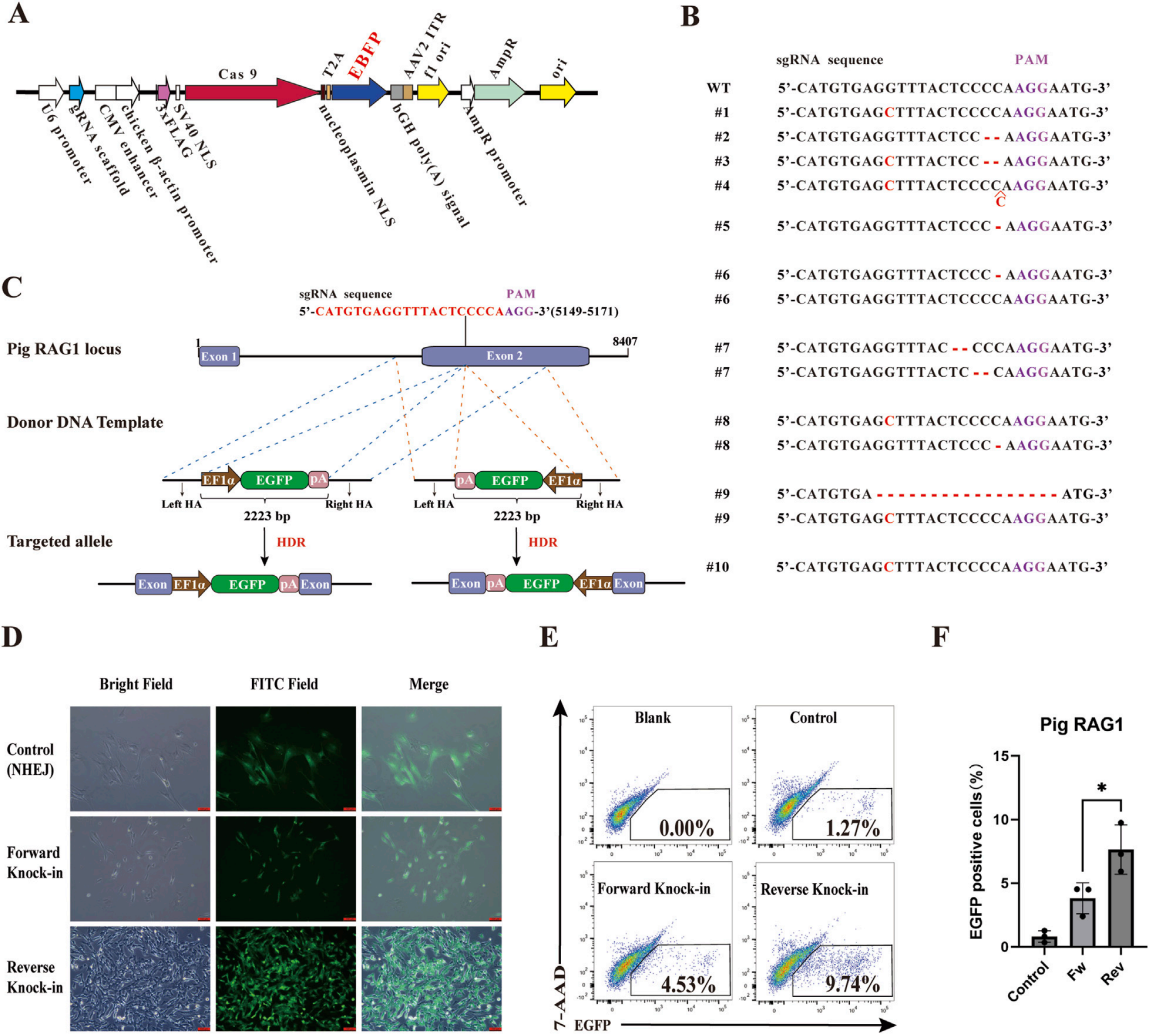
Comparison of HDR efficiency between forward and reverse knock-in methods in the pig genome. **(A)** The plasmid map of the *RAG1*-targeting plasmid PX458-EBFP-sgRNA. **(B)** Analysis of *RAG1* genotyping and knockout efficiency in PFFs. **(C)** Schematic diagrams of donor template design (HAs-EF1α-EGFP-polyA) for both forward and reverse knock-in at sgRNA site in the pig *RAG1* locus. EF1α: elongation factor 1 alpha promoter; pA: bovine growth hormone polyadenylation signal (bGH polyA signal). **(D)** Observation of EGFP^+^ monoclonal colonies by fluorescence microscopy. **(E)** Flow Cytometry results showing EGFP^+^ cell percentages in forward and reverse knock-in groups. **(F)** Quantitative comparison of HDR efficiencies between forward and reverse knock-in groups. Statistical significance was determined using Student’s t-test: *p < 0.05, n = 3.

To further explore whether this reverse donor orientation strategy is broadly applicable across different genomic loci and livestock species, we extended our investigation to the pig *RAG1* gene. A specific sgRNA was designed, and the corresponding PX458-sgRNA plasmid was constructed. Subsequently, the EGFP gene in the PX458-sgRNA plasmid was replaced with the EBFP blue fluorescent reporter to construct the *RAG1*-targeting plasmid PX458-EBFP-sgRNA (Figure 3A). This plasmid was transfected into Wuzhishan PFFs to assess knockout efficiency. All 10 examined clones exhibited mutations in the *RAG1* locus (Figure 3B), confirming efficient CRISPR/Cas9-mediated editing and validating the suitability of this site for targeted integration. Based on this result, we constructed corresponding forward and reverse donor templates (HAs-EF1α-EGFP-polyA) (Figure 3C). The PX458-EBFP-sgRNA plasmid was co-transfected with either the forward or reverse donor template into PFFs. PX458-EBFP-sgRNA plasmid co-transfected with EF1α-EGFP-polyA fragments lacing homology arms served as negative controls. Cells were transfected with the donor template alone as a blank control. In each well of a 6-well plate, 5000 EBFP and EGFP double-positive cells were sorted. After 8 days of culture, EGFP^+^ monoclonal colonies were observed under a fluorescence microscope (Figure 3D). Following 14 days of culture, flow cytometric analysis revealed that the HDR efficiency was significantly higher in the reverse-integration groups compared to the forward-integration groups (Figures 3E,F).

These results demonstrate that reverse donor orientation enables superior knock-in efficiency not only at a different genomic locus but also in a different species.

### 3.3 More hBChE positive goat fibroblast cell clones were produced by reverse knock-in method

**FIGURE 4:**
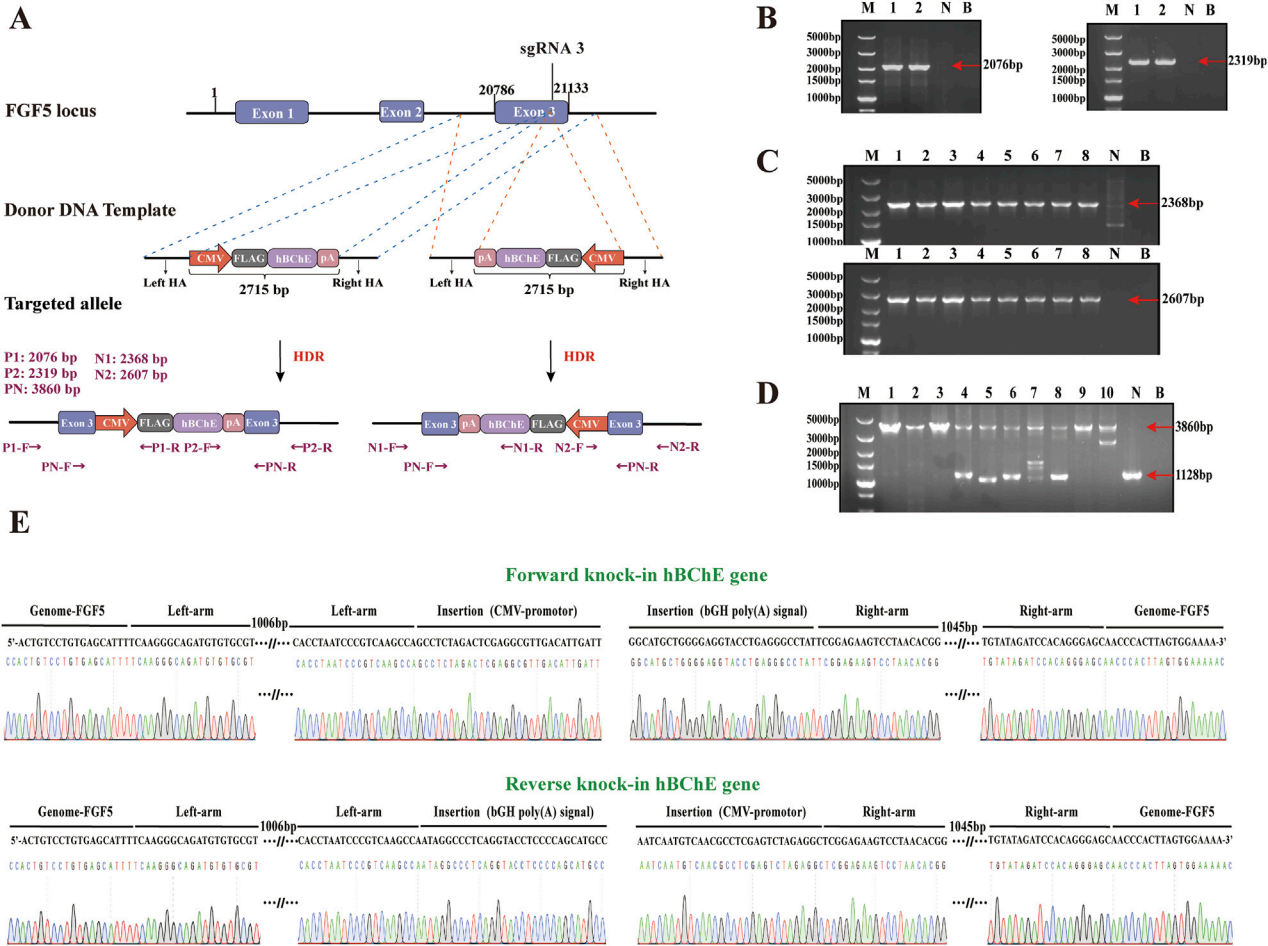
Establishment and genotyping of goat fibroblast clones with hBChE integration. **(A)** Schematic diagrams of donor templates for forward and reverse knock-in of the hBChE gene at the sgRNA3 site of the goat *FGF5* locus. **(B)** PCR identification of forward knock-in clones using primers P1, P2 and PN. Lane M: DNA Marker; Lanes 1–2: positive clones; Lane N: negative control; Lane B blank control. **(C)** PCR identification of reverse knock-in clones using primers N1, N2, and PN. Lane M: Marker; Lanes 1–8: positive clones; Lanes N and B negative/blank controls. **(D)** Genotyping results showing homozygous and heterozygous hBChE integration. Lanes 1–3: homozygous reverse; Lanes 4–8: heterozygous reverse; Lane 9: homozygous forward; Lane 10: heterozygous forward. **(E)** Sanger sequencing confirmation of positive clones.

To generate goat fibroblast cell clones with either forward or reverse knock-in of the hBChE gene, we constructed forward and reverse donor templates (HAs-CMV-FLAG-hBChE-polyA) based on sgRNA3 targeting the *FGF5* locus (Figure 4A). PX458-sgRNA plasmids were co-transfected with either the forward or reverse donor templates into gFCs. After transfection. EGFP^+^ single cells were isolated via flow cytometry and cultured for 12 days.

A total of 190 cell clones derived from forward knock-in were screened by PCR using P1, P2, and PN primers, resulting in the identification of 2 hBChE^+^ clones (positive rate: 1.05%) (Figure 4B). Among these, 1 clone was homozygous and 1 clone was heterozygous (each accounting for 0.53%) (Figure 4D). Meanwhile, 245 cell clones derived from reverse knock-in were screened using N1, N2, and PN primers, yielding 8 hBChE^+^ clones (positive rate: 3.27%) (Figure 4C). Of these, 3 clones were homozygous clones (1.22%) and 5 clones were heterozygous (2.04%) (Figure 4D). All PCR products were validated by Sanger sequencing to confirm precise genome integration (Figure 4E). Taken together, these results demonstrate that the reverse knock-in method generated a greater number of hBChE^+^ cell clones compared to the forward knock-in method.

### 3.4 Cells in reverse knock-in group showed higher expression of rhBChE and the OPPs resistance

**FIGURE 5:**
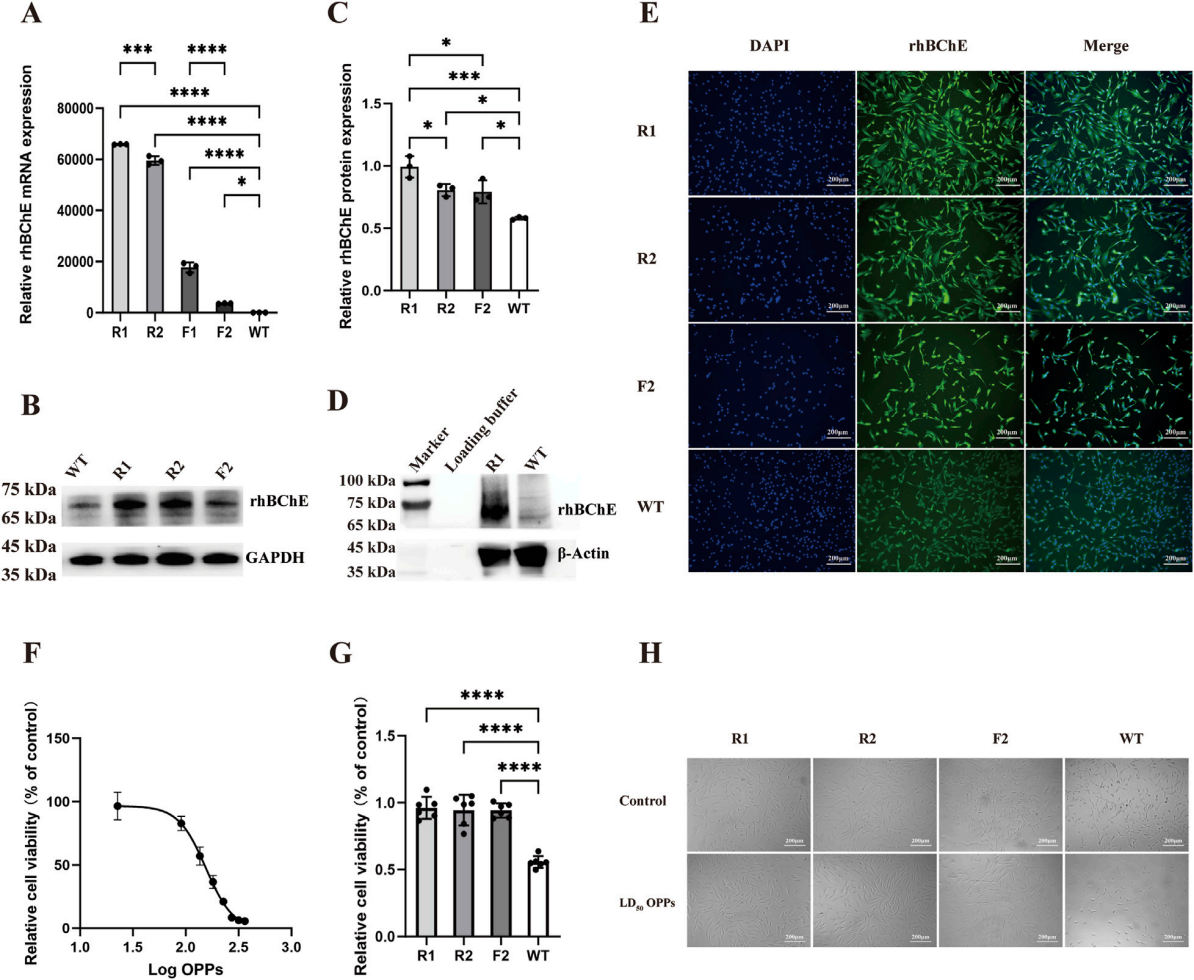
rhBChE expression and OPPs resistance in edited goat fibroblast clones. **(A)** Quantitative RT-PCR analysis of rhBChE mRNA levels in forward and reverse knock-in cell lines. Statistical significance determined by one-way ANOVA: *p < 0.05, ***p < 0.001, ****p < 0.0001, n = 3. **(B)** Western blot detection of rhBChE protein expression levels; GAPDH served as a loading control. **(C)** Quantification of rhBChE protein expression levels. Statistical analysis by one-way ANOVA: *p < 0.05, ***p < 0.001, n = 3. **(D)** Western blot detection of FLAG protein expression levels; β-Actin served as a loading control. **(E)** Immunofluorescence staining showing rhBChE protein expression in edited cell lines. **(F)** Determination of the LD_50_ of OPPs for WT cells: 154.9 μM. **(G)** CCK-8 assay showing cell viability after OPPs treatment. Statistical analysis by one-way ANOVA: ****p < 0.0001, n = 6. **(H)** Cell morphology of hBChE^+^ cell clones cultured in complete medium supplemented with 154.9 μM OPPs for 24 h.

The rhBChE expression at the mRNA level was detected in 4 cell lines: one reverse knock-in homozygous line (R1), one reverse knock-in heterozygous line (R2), one forward knock-in homozygous line (F1), and one forward knock-in heterozygous line (F2). Quantitative real-time PCR analysis demonstrated that the expression levels of rhBChE mRNA in the reverse knock-in cell lines (R1 and R2) were significantly higher than those in the forward knock-in cell lines (F1 and F2) (Figure 5A) At the protein level, rhBChE expression was analyzed by immunofluorescence and western blotting. Due to the poor viability of the F1 cell line, only R1, R2 and F2 were assessed for protein expression. Immunofluorescence staining revealed enhanced rhBChE protein expression in all 3 cell lines (Figure 5E). Western blotting analysis demonstrated that rhBChE protein levels were significantly elevated in R1, R2 and F2 compared to WT gFCs, consistent with the mRNA expression results (Figures 5B,C). To specifically detect the rhBChE and avoid potential interference from endogenous BChE in gFCs, an antibody targeting the FLAG tag was used. Western blotting confirmed robust expression of FLAG-tagged rhBChE in R1-positive cells (Figure 5D).

To evaluate the resistance of these cell lines to OPPs, the LD_50_ for OPPs in WT gFCs was determined to be 154.9 μM (Figure 5F). Subsequently, four cell lines (R1, R2, F2 and WT) were individually cultured in complete medium supplemented with OPPs at 154.9 μM for 24 h. Observation under a microscope showed with OPPs that the hBChE^+^ cell lines maintained their activity and showed no significant alterations in cell morphology (Figure 5H). Furthermore, according to the results of the CCK-8 assay, the hBChE^+^ cell lines demonstrated significantly enhanced resistance to OPPs compared to WT gFCs (Figure 5G).

### 3.5 Off-target detection

**FIGURE 6:**
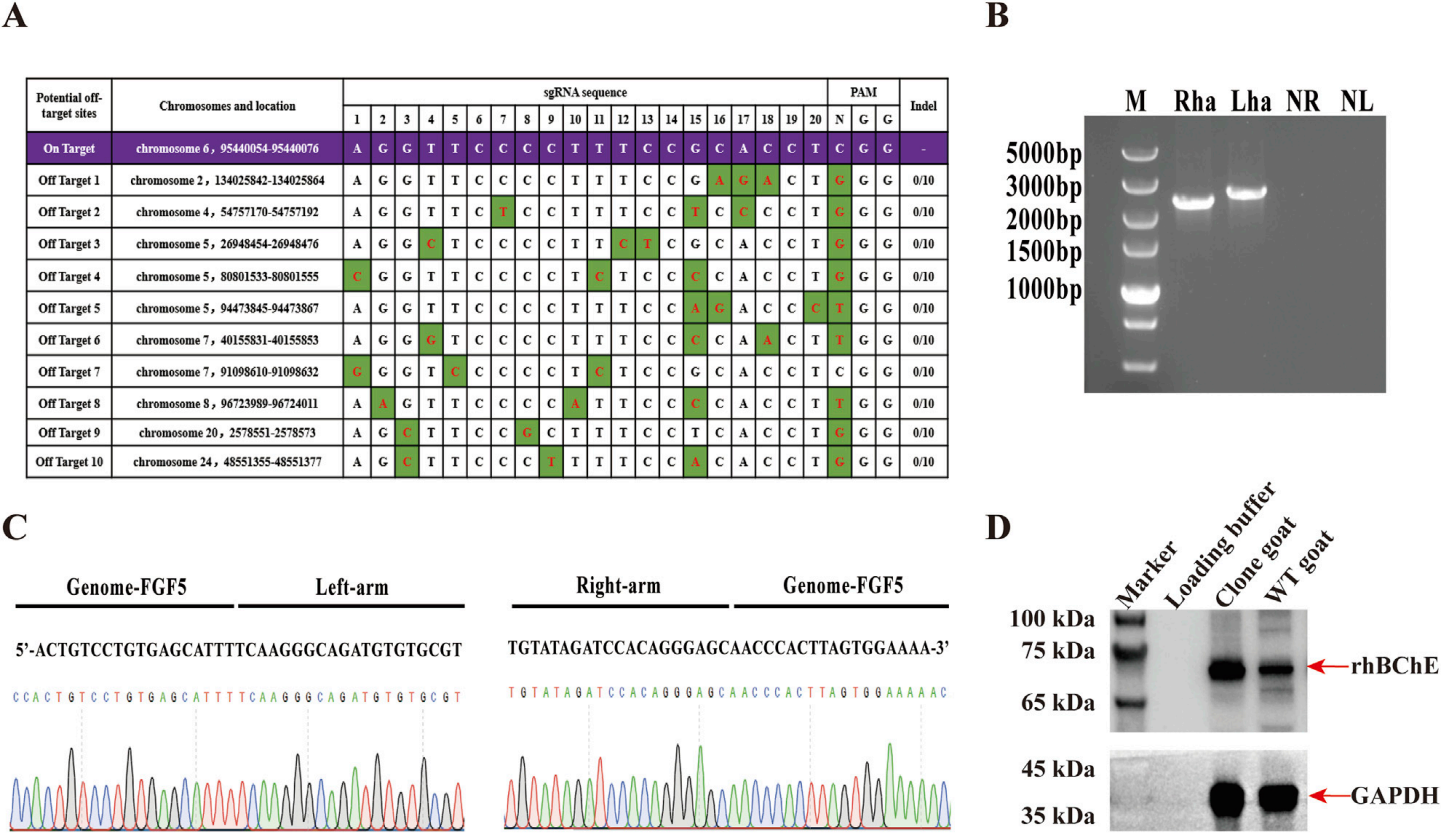
Generation and characterization of cloned goats expressing rhBChE. **(A)** Predicted off-target sites identified using Cas-OFFinder software and validated by sanger sequencing. Variants highlighted in green. **(B)** PCR confirmation of hBChE integration in cloned goat using N1 and N2 primers. Lanes Rha and Lha: cloned goat; NR and NL: WT goat controls. **(C)** Sanger sequencing results confirming hBChE integration in the cloned goat genome. **(D)** Western blot analysis of rhBChE protein expression in cloned goat skin tissue.

To check the potential off-target effects in the hBChE^+^ cell lines, we identified a total of 10 potential off-target sites using Cas-OFFinder software. These sites were amplified by PCR and subjected to Sanger sequencing. The sequencing results were compared with the corresponding NCBI reference sequences. No nucleotide mutations were observed at any of the predicted off-target loci, indicating the absence of off-target effects in the edited cells (Figure 6A).

### 3.6 Preparation of cloned goats expressing rhBChE using cells in reverse knock-in group

Given the superior cell viability and rhBChE expression in the reverse knock-in hBChE cell lines, we selected a reverse knock-in homozygous clone (R1) for somatic cell nuclear transfer (SCNT) to produce cloned goats. A total of 104 MII-stage oocytes were collected from 10 superovulated Laoshan dairy goats, of which 92 (88.46%) were deemed suitable for SCNT. Following electrofusion, 66 reconstructed embryos were obtained and subsequently were transferred into 4 female recipients. Pregnancy diagnosis confirmed that one recipient became pregnant, and a goat was born. Genomic DNA extracted from the blood of the goat was subjected to PCR using N1 and N2 primers, confirming the integration of the hBChE gene (Figure 6B). Sanger sequencing further validated the precise integration of the hBChE gene into the cloned goat genome (Figure 6C). To assess rhBChE protein expression in the cloned goat, total protein was extracted from the skin tissue and analyzed by western blotting. The results demonstrated high-level expression of the rhBChE protein in the cloned goat (Figure 6D).

## 4 Discussion

CRISPR/Cas9 genome editing technology facilitates the efficient and precise integration of exogenous genes into specific genomic loci, providing powerful support for the development of animal bioreactors and the production of recombinant proteins. The high-level expression of exogenous recombinant proteins in mammalian cells and transgenic animal bioreactors is critical for biotechnological applications. Our study demonstrated that reverse knock-in hBChE cell lines showed significantly higher expression levels of rhBChE compared to forward knock-in lines. This difference may be attributed to the presence of transcriptional terminators: specifically, the endogenous *FGF5* terminator and the bGH poly(A) signal in the rhBChE expression cassette.

Previous researches have revealed that a single transcription terminator can terminate adjacent genes transcribed in opposite orientations across various organisms, including *Escherichia coli*, *Saccharomyces cerevisiae* and mammals (Postle and Good, 1985; Uwimana et al., 2017; Deykin et al., 2019; Ju et al., 2019). Furthermore, introducing an insulator or transcription termination sequence upstream of a transgene promoter can greatly enhance transcriptional efficiency and stability (Huang et al., 2007; Deykin et al., 2019).

To further investigate the effect of bidirectional transcriptional termination, we conducted two plasmids: p-mCherry and p-mCherry-EGFP (Supplementary Figure S1A). In p-mCherry-EGFP plasmid, the bGH poly(A) signal was inserted between two fluorescent reporter genes, mCherry and EGFP. Flow cytometry analysis showed that cell transfected with p-mCherry-EGFP expressed both EGFP and mCherry fluorescence signals, which is consistent with the possibility that the bGH poly(A) signal possesses bidirectional transcription termination activity (Supplementary Figure S1B).

Based on these findings, we propose that the bGH poly(A) signal in the reverse knock-in rhBChE expression vector may function as a bidirectional transcriptional terminator: it not only ensures proper 3′end processing of the rhBChE transcript but also terminates upstream transcription from the endogenous *FGF5* gene, thereby insulating the transgene from read-through interference. In contrast, in forward knock-in cells, the lack of a compatible terminator downstream of *FGF5* in the same transcriptional orientation permits transcriptional read-through into the rhBChE expression cassette, resulting in transcriptional interference that may impair transgene expression. Furthermore, we speculate that the endogenous *FGF5* terminator itself may also possess bidirectional activity. In the reverse-integration design, this terminator could reduce interference with the CMV promoter driving rhBChE expression, thereby contributing to enhanced transgene stability and expression.

Notably, the higher HDR efficiency observed in the reverse knock-in cell lines was not limited to a single genomic locus or cell type, but was reproducible across diverse genomic targets and livestock species. This effect appears independent of the specific promoter, transgene, or host cell type, suggesting a broadly applicable mechanism. We hypothesize that the bidirectional termination activity of both the endogenous *FGF5* terminator and the bGH pA signal mitigates transcriptional interference, thereby reducing transcriptional stress, enhancing cellular viability, and promoting the survival of HDR-positive cells. This selective advantage likely increases the yield of viable, correctly edited clones, contributing to the elevated HDR efficiency observed in the reverse configuration.

In addition to successfully establishing positive cell lines, we observed a significant upregulation of rhBChE mRNA levels, yet the corresponding increase in rhBChE protein expression was limited. This discrepancy between transcript abundance and protein output reflects the multi-layered regulation inherent in gene expression. It is well established that mRNA levels account for only 40%–60% of the variation in protein abundance across cellular contexts (Schwanhäusser et al., 2011; Vogel and Marcotte, 2012). The remaining variability is largely driven by post-transcriptional mechanisms, particularly translation efficiency and protein degradation rates. As a result, high mRNA levels do not always translate into proportional protein accumulation, due to inefficient translation or rapid turnover of newly synthesized proteins. From an evolutionary standpoint, protein abundances are more conserved across species than mRNA levels (Laurent et al., 2010; Weiss et al., 2010), indicating that post-transcriptional regulatory networks have evolved to stabilize the proteome and buffer transcriptional fluctuations. Moreover, rhBChE is a complex secreted glycoprotein that requires proper folding, glycosylation, and assembly in the endoplasmic reticulum (ER). Its overexpression can exceed the ER’s processing capacity, leading to ER stress and activation of quality control mechanisms such as ER-associated degradation (ERAD) (Vogel and Marcotte, 2012). Consequently, the translational and secretory machinery may become saturated, limiting functional protein yield even as mRNA levels continue to rise.

In summary, our study demonstrated that although both forward and reverse knock-in hBChE cell lines were capable of efficiently expressing rhBChE protein and exhibited significant OPPs resistance, the reverse knock-in cell lines showed significantly higher HDR efficiency and greater cellular stability compared to the forward knock-in cell lines. Futhermore, we successfully generated a goat overexpression rhBChE using the reverse knock-in hBChE cell line by SCNT technology. These results offer important insights into how CRISPR/Cas9 gene editing technology can be optimized for development of efficient and reliable transgenic animal bioreactors.

## 5 Limitations

This study has limitations. First, the biological mechanisms responsible for the enhanced HDR efficiency observed with reverse knock-in donor templates remain incompletely understood. Second, although rhBChE-expressing cell lines and a cloned goat were successfully generated, several critical translational aspects remain unexplored. In particular, we did not quantity rhBChE production levels in mammary gland secretions, assess long-term stability of transgene expression, or analyze post-translational modifications of the recombinant protein. These factors could significantly influence both the yield and therapeutic quality of the produced enzyme and should be addressed in future studies through longitudinal *in vivo* assessment and biochemical characterization.

## Data Availability

The original contributions presented in the study are included in the article/Supplementary Material, further inquiries can be directed to the corresponding authors.

## References

[B1] Adiguzel C. Iqbal O. Demir M. Fareed J. (2009). European community and US-FDA approval of recombinant human antithrombin produced in genetically altered goats. Clin. Appl. Thromb. Hemost. 15 (6), 645–651. 10.1177/1076029609339748 19850586

[B2] Allen F. Crepaldi L. Alsinet C. Strong A. J. Kleshchevnikov V. De Angeli P. (2018). Predicting the mutations generated by repair of Cas9-induced double-strand breaks. Nat. Biotechnol. 37, 64–72. 10.1038/nbt.4317 30480667 PMC6949135

[B3] Amiri Yekta A. Dalman A. Eftekhari-Yazdi P. Sanati M. H. Shahverdi A. H. Fakheri R. (2013). Production of transgenic goats expressing human coagulation factor IX in the mammary glands after nuclear transfer using transfected fetal fibroblast cells. Transgenic Res. 22 (1), 131–142. 10.1007/s11248-012-9634-y 22869287

[B4] Aoyama M. Agari K. Sun-Wada G. H. Futai M. Wada Y. (2005). Simple and straightforward construction of a mouse gene targeting vector using *in vitro* transposition reactions. Nucleic Acids Res. 33 (5), e52. 10.1093/nar/gni055 15784610 PMC1069132

[B5] Auer T. O. Duroure K. De Cian A. Concordet J. P. Del Bene F. (2014). Highly efficient CRISPR/Cas9-mediated knock-in in zebrafish by homology-independent DNA repair. Genome Res. 24 (1), 142–153. 10.1101/gr.161638.113 24179142 PMC3875856

[B6] Broomfield C. A. Maxwell D. M. Solana R. P. Castro C. A. Finger A. V. Lenz D. E. (1991). Protection by butyrylcholinesterase against organophosphorus poisoning in nonhuman primates. J. Pharmacol. Exp. Ther. 259 (2), 633–638. 10.1016/s0022-3565(25)20479-7 1941611

[B7] Chakrabarti A. M. Henser-Brownhill T. Monserrat J. Poetsch A. R. Luscombe N. M. Scaffidi P. (2019). Target-specific precision of CRISPR-mediated genome editing. Mol. Cell 73 (4), 699–713.e6. 10.1016/j.molcel.2018.11.031 30554945 PMC6395888

[B8] Chu V. T. Weber T. Wefers B. Wurst W. Sander S. Rajewsky K. (2015). Increasing the efficiency of homology-directed repair for CRISPR-Cas9-induced precise gene editing in mammalian cells. Nat. Biotechnol. 33 (5), 543–548. 10.1038/nbt.3198 25803306

[B9] Cui C. Song Y. Liu J. Ge H. Li Q. Huang H. (2015). Gene targeting by TALEN-induced homologous recombination in goats directs production of β-lactoglobulin-free, high-human lactoferrin milk. Sci. Rep. 5, 10482. 10.1038/srep10482 25994151 PMC5386245

[B10] Deykin A. Tikhonov M. Kalmykov V. Korobko I. Georgiev P. Maksimenko O. (2019). Transcription termination sequences support the expression of transgene product secreted with milk. Transgenic Res. 28 (3-4), 401–410. 10.1007/s11248-019-00122-9 30919251

[B11] Dierks C. Mömke S. Philipp U. Distl O. (2013). Allelic heterogeneity of FGF5 mutations causes the long-hair phenotype in dogs. Anim. Genet. 44 (4), 425–431. 10.1111/age.12010 23384345

[B12] Drögemüller C. Rüfenacht S. Wichert B. Leeb T. (2007). Mutations within the FGF5 gene are associated with hair length in cats. Anim. Genet. 38 (3), 218–221. 10.1111/j.1365-2052.2007.01590.x 17433015

[B13] Goldman I. L. Georgieva S. G. Gurskiy Y. G. Krasnov A. N. Deykin A. V. Popov A. N. (2012). Production of human lactoferrin in animal milk^1^This article is part of a special issue entitled lactoferrin and has undergone the Journal's usual peer review process. Biochem. Cell Biol. 90 (3), 513–519. 10.1139/o11-088 22360490

[B14] Gui T. Zhang M. Chen J. Zhang Y. Zhou N. Zhang Y. (2012). *In vitro* evaluation of a mammary gland specific expression vector encoding recombinant human lysozyme for development of transgenic dairy goat embryos. Biotechnol. Lett. 34 (8), 1445–1452. 10.1007/s10529-012-0930-7 22526423

[B15] Hennighausen L. (1990). The mammary gland as a bioreactor: production of foreign proteins in milk. Protein Expr. Purif. 1 (1), 3–8. 10.1016/1046-5928(90)90037-y 2152181

[B16] Higgins C. A. Petukhova L. Harel S. Ho Y. Y. Drill E. Shapiro L. (2014). FGF5 is a crucial regulator of hair length in humans. Proc. Natl. Acad. Sci. U. S. A. 111 (29), 10648–10653. 10.1073/pnas.1402862111 24989505 PMC4115575

[B17] Houdebine L. M. (2009). Production of pharmaceutical proteins by transgenic animals. Comp. Immunol. Microbiol. Infect. Dis. 32 (2), 107–121. 10.1016/j.cimid.2007.11.005 18243312 PMC7112688

[B18] Housley D. J. Venta P. J. (2006). The long and the short of it: evidence that FGF5 is a major determinant of canine 'hair'-itability. Anim. Genet. 37 (4), 309–315. 10.1111/j.1365-2052.2006.01448.x 16879338

[B19] Hu X. Hao F. Li X. Xun Z. Gao Y. Ren B. (2021). Generation of VEGF knock-in cashmere goat *via* the CRISPR/Cas9 system. Int. J. Biol. Sci. 17 (4), 1026–1040. 10.7150/ijbs.55559 33867826 PMC8040296

[B20] Huang Y. J. Huang Y. Baldassarre H. Wang B. Lazaris A. Leduc M. (2007). Recombinant human butyrylcholinesterase from milk of transgenic animals to protect against organophosphate poisoning. Proc. Natl. Acad. Sci. U. S. A. 104 (34), 13603–13608. 10.1073/pnas.0702756104 17660298 PMC1934339

[B21] Johnson G. Moore S. W. (2012). Why has butyrylcholinesterase been retained? Structural and functional diversification in a duplicated gene. Neurochem. Int. 61 (5), 783–797. 10.1016/j.neuint.2012.06.016 22750491

[B22] Ju X. Li D. Liu S. (2019). Full-length RNA profiling reveals pervasive bidirectional transcription terminators in bacteria. Nat. Microbiol. 4 (11), 1907–1918. 10.1038/s41564-019-0500-z 31308523 PMC6814526

[B23] Kehler J. S. David V. A. Schäffer A. A. Bajema K. Eizirik E. Ryugo D. K. (2007). Four independent mutations in the feline fibroblast growth factor 5 gene determine the long-haired phenotype in domestic cats. J. Hered. 98 (6), 555–566. 10.1093/jhered/esm072 17767004 PMC3756544

[B24] Laurent J. M. Vogel C. Kwon T. Craig S. A. Boutz D. R. Huse H. K. (2010). Protein abundances are more conserved than mRNA abundances across diverse taxa. Proteomics 10 (23), 4209–4212. 10.1002/pmic.201000327 21089048 PMC3113407

[B25] Legrand R. Tiret L. Abitbol M. (2014). Two recessive mutations in FGF5 are associated with the long-hair phenotype in donkeys. Genet. Sel. Evol. 46 (1), 65. 10.1186/s12711-014-0065-5 25927731 PMC4175617

[B26] Lenz D. E. Maxwell D. M. Koplovitz I. Clark C. R. Capacio B. R. Cerasoli D. M. (2005). Protection against soman or VX poisoning by human butyrylcholinesterase in guinea pigs and cynomolgus monkeys. Chem. Biol. Interact. 157-158, 205–210. 10.1016/j.cbi.2005.10.025 16289064

[B27] Li W. R. Liu C. X. Zhang X. M. Chen L. Peng X. R. He S. G. (2017). CRISPR/Cas9-mediated loss of FGF5 function increases wool staple length in sheep. Febs J. 284 (17), 2764–2773. 10.1111/febs.14144 28631368

[B28] Li H. Li Z. Xiao N. Su X. Zhao S. Zhang Y. (2019). Site-specific integration of rotavirus VP6 gene in rabbit β-casein locus by CRISPR/Cas9 system. Vitro Cell Dev. Biol. Anim. 55 (8), 586–597. 10.1007/s11626-019-00382-z 31367859

[B29] Li Y. Lian D. Wang J. Zhao Y. Li Y. Liu G. (2023). MDM2 antagonists promote CRISPR/Cas9-mediated precise genome editing in sheep primary cells. Mol. Ther. Nucleic Acids 31, 309–323. 10.1016/j.omtn.2022.12.020 36726409 PMC9883270

[B30] Ma M. Zhuang F. Hu X. Wang B. Wen X. Z. Ji J. F. (2017). Efficient generation of mice carrying homozygous double-floxp alleles using the Cas9-Avidin/Biotin-donor DNA system. Cell Res. 27 (4), 578–581. 10.1038/cr.2017.29 28266543 PMC5385615

[B31] Maga E. A. Shoemaker C. F. Rowe J. D. Bondurant R. H. Anderson G. B. Murray J. D. (2006). Production and processing of milk from transgenic goats expressing human lysozyme in the mammary gland. J. Dairy Sci. 89 (2), 518–524. 10.3168/jds.S0022-0302(06)72114-2 16428620

[B32] Mann T. M. Price M. E. Whitmore C. L. Perrott R. L. Laws T. R. McColm R. R. (2018). Bioscavenger is effective as a delayed therapeutic intervention following percutaneous VX poisoning in the guinea-pig. Toxicol. Lett. 293, 198–206. 10.1016/j.toxlet.2017.11.029 29183815

[B33] Postle K. Good R. F. (1985). A bidirectional rho-independent transcription terminator between the *E. coli* tonB gene and an opposing gene. Cell 41 (2), 577–585. 10.1016/s0092-8674(85)80030-1 2985285

[B34] Reed B. A. Sabourin C. L. Lenz D. E. (2017). Human butyrylcholinesterase efficacy against nerve agent exposure. J. Biochem. Mol. Toxicol. 31 (5), e21886. 10.1002/jbt.21886 28225154

[B35] Rice H. Mann T. M. Armstrong S. J. Price M. E. Green A. C. Tattersall J. E. H. (2016). The potential role of bioscavenger in the medical management of nerve-agent poisoned casualties. Chem. Biol. Interact. 259 (Pt B), 175–181. 10.1016/j.cbi.2016.04.038 27144491

[B36] Richardson C. D. Ray G. J. DeWitt M. A. Curie G. L. Corn J. E. (2016). Enhancing homology-directed genome editing by catalytically active and inactive CRISPR-Cas9 using asymmetric donor DNA. Nat. Biotechnol. 34 (3), 339–344. 10.1038/nbt.3481 26789497

[B37] Rosenberg Y. Saxena A. (2020). Acetylcholinesterase inhibition resulting from exposure to inhaled OP can be prevented by pretreatment with BChE in both macaques and minipigs. Neuropharmacology 174, 108150. 10.1016/j.neuropharm.2020.108150 32442543 PMC7365266

[B38] Safari F. Farajnia S. Ghasemi Y. Zarghami N. (2017). New developments in CRISPR technology: improvements in specificity and efficiency. Curr. Pharm. Biotechnol. 18 (13), 1038–1054. 10.2174/1389201019666180209120533 29424307

[B39] Saxena A. Sun W. Dabisch P. A. Hulet S. W. Hastings N. B. Jakubowski E. M. (2011a). Pretreatment with human serum butyrylcholinesterase alone prevents cardiac abnormalities, seizures, and death in göttingen minipigs exposed to sarin vapor. Biochem. Pharmacol. 82 (12), 1984–1993. 10.1016/j.bcp.2011.09.019 21968035

[B40] Saxena A. Sun W. Fedorko J. M. Koplovitz I. Doctor B. P. (2011b). Prophylaxis with human serum butyrylcholinesterase protects Guinea pigs exposed to multiple lethal doses of soman or VX. Biochem. Pharmacol. 81 (1), 164–169. 10.1016/j.bcp.2010.09.007 20846507

[B41] Schmidt C. (2006). Belated approval of first recombinant protein from animal. Nat. Biotechnol. 24 (8), 877. 10.1038/nbt0806-877 16900113

[B42] Schwanhäusser B. Busse D. Li N. Dittmar G. Schuchhardt J. Wolf J. (2011). Global quantification of mammalian gene expression control. Nature 473 (7347), 337–342. 10.1038/nature10098 21593866

[B43] Scully R. Panday A. Elango R. Willis N. A. (2019). DNA double-strand break repair-pathway choice in somatic mammalian cells. Nat. Rev. Mol. Cell Biol. 20 (11), 698–714. 10.1038/s41580-019-0152-0 31263220 PMC7315405

[B44] Uwimana N. Collin P. Jeronimo C. Haibe-Kains B. Robert F. (2017). Bidirectional terminators in *Saccharomyces cerevisiae* prevent cryptic transcription from invading neighboring genes. Nucleic Acids Res. 45 (11), 6417–6426. 10.1093/nar/gkx242 28383698 PMC5499651

[B45] Varga L. Farkas H. (2011). rhC1INH: a new drug for the treatment of attacks in hereditary angioedema caused by C1-inhibitor deficiency. Expert Rev. Clin. Immunol. 7 (2), 143–153. 10.1586/eci.11.5 21426252

[B46] Vogel C. Marcotte E. M. (2012). Insights into the regulation of protein abundance from proteomic and transcriptomic analyses. Nat. Rev. Genet. 13 (4), 227–232. 10.1038/nrg3185 22411467 PMC3654667

[B47] Wang J. Yang P. Tang B. Sun X. Zhang R. Guo C. (2008). Expression and characterization of bioactive recombinant human alpha-lactalbumin in the milk of transgenic cloned cows. J. Dairy Sci. 91 (12), 4466–4476. 10.3168/jds.2008-1189 19038921

[B48] Wang T. Wei J. J. Sabatini D. M. Lander E. S. (2014). Genetic screens in human cells using the CRISPR-Cas9 system. Science 343 (6166), 80–84. 10.1126/science.1246981 24336569 PMC3972032

[B49] Wang X. Yu H. Lei A. Zhou J. Zeng W. Zhu H. (2015). Generation of gene-modified goats targeting MSTN and FGF5 *via* zygote injection of CRISPR/Cas9 system. Sci. Rep. 5, 13878. 10.1038/srep13878 26354037 PMC4564737

[B50] Wang X. Cai B. Zhou J. Zhu H. Niu Y. Ma B. (2016). Disruption of FGF5 in cashmere goats using CRISPR/Cas9 results in more secondary hair follicles and longer fibers. PLoS One 11 (10), e0164640. 10.1371/journal.pone.0164640 27755602 PMC5068700

[B51] Wang J. H. Wu S. J. Li Y. Zhao Y. Liu Z. M. Deng S. L. (2023). Improving the efficiency of precise genome editing with CRISPR/Cas9 to generate goats overexpressing human butyrylcholinesterase. Cells 12 (14), 1818. 10.3390/cells12141818 37508483 PMC10378061

[B52] Weiss M. Schrimpf S. Hengartner M. O. Lercher M. J. von Mering C. (2010). Shotgun proteomics data from multiple organisms reveals remarkable quantitative conservation of the eukaryotic core proteome. Proteomics 10 (6), 1297–1306. 10.1002/pmic.200900414 20077411

[B53] Xing S. Li Q. Xiong B. Chen Y. Feng F. Liu W. (2021). Structure and therapeutic uses of butyrylcholinesterase: application in detoxification, alzheimer's disease, and fat metabolism. Med. Res. Rev. 41 (2), 858–901. 10.1002/med.21745 33103262

[B54] Xu H. T. Fan B. L. Yu S. Y. Huang Y. H. Zhao Z. H. Lian Z. X. (2007). Construct synthetic gene encoding artificial spider dragline silk protein and its expression in milk of transgenic mice. Anim. Biotechnol. 18 (1), 1–12. 10.1080/10495390601091024 17364439

[B55] Zhang R. Rao M. Li C. Cao J. Meng Q. Zheng M. (2009). Functional recombinant human anti-HAV antibody expressed in milk of transgenic mice. Transgenic Res. 18 (3), 445–453. 10.1007/s11248-008-9241-0 19130282 PMC7089081

[B56] Zhang R. Cui D. Wang H. Li C. Yao X. Zhao Y. (2012). Functional recombinant human anti-HBV antibody expressed in milk of transgenic mice. Transgenic Res. 21 (5), 1085–1091. 10.1007/s11248-012-9589-z 22286336

[B57] Zhang R. Li Y. Jia K. Xu X. Li Y. Zhao Y. (2020). Crosstalk between androgen and Wnt/β-catenin leads to changes of wool density in FGF5-knockout sheep. Cell Death Dis. 11 (5), 407. 10.1038/s41419-020-2622-x 32472005 PMC7260202

