# Supplementary material for: Optimization of donor structure enhances the generation of cloned goats with high expression of human butyrylcholinesterase by CRISPR/Cas9
Source: Front Bioeng Biotechnol. 2025 Nov 10;13:1633553. doi: 10.3389/fbioe.2025.1633553 (PMC12641187; doi:10.3389/fbioe.2025.1633553)
Supplement: Supplementary file 1 [file Supplementaryfile1.docx]

Supplementary Material

# Supplementary Figure and Tables

## Supplementary Figure

**Supplementary Figure S1.** Experimental verification of the bidirectional transcription termination ability of the bGH poly(A) signal.


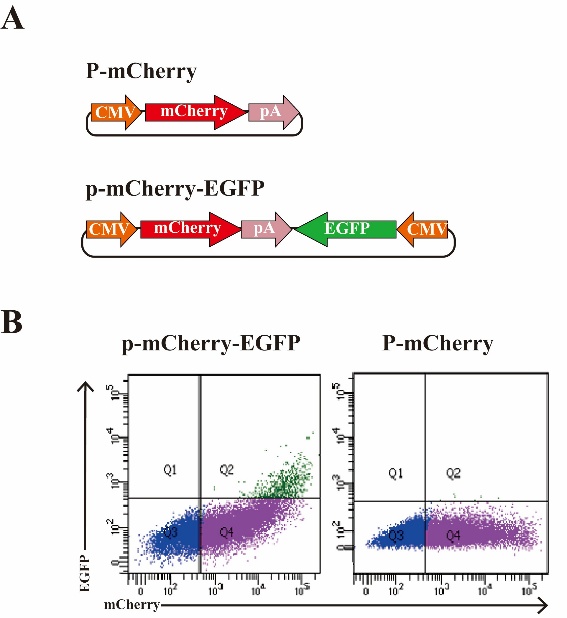


## Supplementary Tables

**Supplementary Table S1.** Primer sequences used for identifying *RAG1* knockout cell clones.

| Name | Sequences (5’-3’) |
| --- | --- |
| Rag1-F | CTTGTCCTTATTGCTCCCAGGT |
| Rag2-R | AGATTTCACAAAGTGCGCCG |

**Supplementary Table S2.** Primer sequences used for identifying hBChE-positive cell clones.

| Name | Sequences (5’-3’) |
| --- | --- |
| P1-F | TCAGTACAGCACACTGAGCC |
| P1-R | AGTCTCCGTCGTGGTCCTTA |
| P2-F | GGGTCTCTAAAGCTCGCTGA |
| P2-R | TCTTCTGCTCTCCTGTTCTGC |
| N1-F | ATGGTCCTCCCTGAAATTGTGAA |
| N1-R | GTGGCCAGAATGGATGGGAG |
| N2-F | GGGCGGAGTTGTTACGACAT |
| N2-R | TGCTCTCCTGTTCTGCAGTTT |
| PN-F | ATCTGCCTGAAGTGTTGCTAT |
| PN-R | GCTGAAGCTGTGTCCAAGAAG |

**Supplementary Table S3.** Primer sequences used for detecting the rhBChE mRNA expression.

| Name | Sequences (5’-3’) |
| --- | --- |
| Q1-F | ACAGTTTTTGGTGGCACGGT |
| Q1-R | AGGTCAGTGTTTGGGTTCCA |
| βActin-F | AGATGTGGATCAGCAAGCAG |
| βActin-R | CCAATCTCATCTCGTTTTCTG |

**Supplementary Table S4.** Primer sequences used for screening potential off-target sites in hBChE-positive cell lines.

| Name | Sequences (5’-3’) |
| --- | --- |
| Off target 1-F | CCTTACATGGTCGATGCTGG |
| Off target 1-R | TGGTGGCCAACTGTAGACAA |
| Off target 2-F | GCGGGGTAACTGTTTCCAGA |
| Off target 2-R | TTACAGTGTGACCACGGCTC |
| Off target 3-F | AGCTCTGGAAACTCATGCCC |
| Off target 3-R | AGCGAACAAGCGGAGGTAAA |
| Off target 4-F | ATAGCATACACGCCTCTAGCC |
| Off target 4-R | GGAGCAAGTGGTTCACCTCA |
| Off target 5-F | CAAGTGCCGACAAGCTAGGA |
| Off target 5-R | TTCCACAGCATGTAGAGCCG |
| Off target 6-F | ACGACTCCAGTGACCACTCA |
| Off target 6-R | AGTGGGTCTTTGGCATGGAA |
| Off target 7-F | CAAGGTACTCGACCTTCGGG |
| Off target 7-R | TTGTGTGTCATGGGGGCTTT |
| Off target 8-F | TTTGGCAGGCTGGTAGTAGG |
| Off target 8-R | AGTTCGCCAACTTTTGGAAGC |
| Off target 9-F | CAAGCAGGGGCTCTCATTCA |
| Off target 9-R | GCATTCGTAGTCCGTTTGGC |
| Off target 10-F | GGACATATGCCAGTGAATCCG |
| Off target 10-R | AAGGAAAAACGAGCCCAAGTC |
